# Identification of a Missense Variant in *MFSD12* Involved in Dilution of Phaeomelanin Leading to White or Cream Coat Color in Dogs

**DOI:** 10.3390/genes10050386

**Published:** 2019-05-21

**Authors:** Benoit Hédan, Edouard Cadieu, Nadine Botherel, Caroline Dufaure de Citres, Anna Letko, Maud Rimbault, Cord Drögemüller, Vidhya Jagannathan, Thomas Derrien, Sheila Schmutz, Tosso Leeb, Catherine André

**Affiliations:** 1 Institut de Génétique et Développement de Rennes, CNRS-UMR6290, Université de Rennes1, 35000 Rennes, France; edouard.cadieu@univ-rennes1.fr (E.C.); nadine.botherel@univ-rennes1.fr (N.B.); maud.rimbault@gmail.com (M.R.); thomas.derrien@univ-rennes1.fr (T.D.); catherine.andre@univ-rennes1.fr (C.A.); 2 Antagene, 6 allée du Levant, 69890 La Tour de Salvagny, France; cdufauredecitres@antagene.com; 3 Institute of Genetics, University of Bern, 3001 Bern, Swizterland; anna.letko@vetsuisse.unibe.ch (A.L.); cord.droegemueller@vetsuisse.unibe.ch (C.D.); vidhya.jagannathan@vetsuisse.unibe.ch (V.J.); tosso.leeb@vetsuisse.unibe.ch (T.L.); 4 Department of Animal and Poultry Science, University of Saskatchewan, Saskatoon, SK S7N 5A8, Canada; sheila.schmutz@usask.ca

**Keywords:** Dog, *MFSD12*, Locus I, phaeomelanin dilution

## Abstract

White coat color in mammals has been selected several times during the domestication process. Numerous dog breeds are fixed for one form of white coat color that involves darkly pigmented skin. The genetic basis of this color, due to the absence of pigment in the hairs, was suggested to correspond to extreme dilution of the phaeomelanin, by both the expression of only phaeomelanin (locus E) and its extreme dilution (locus I). To go further, we performed genome-wide association studies (GWAS) using a multiple breed approach. The first GWAS, using 34 white dogs and 128 non-white dogs, including White Shepherds, Poodles, Cotons de Tulear and Bichons allowed us to identify two significantly associated loci on the locus E and a novel locus on chromosome 20. A second GWAS using 15 other breeds presenting extreme phaeomelanin dilution confirmed the position of locus I on the chromosome 20 (position 55 Mb *p*_corrected_ = 6 × 10^−13^). Using whole-genome sequencing, we identified a missense variant in the first exon of *MFSD12*, a gene recently identified to be involved in human, mouse and horse pigmentation. We confirmed the role of this variant in phaeomelanin dilution of numerous canine breeds, and the conserved role of *MFSD12* in mammalian pigmentation.

## 1. Introduction

White coat color is a common color in dogs caused by at least two underlying distinct genetic mechanisms: the absence of melanocytes in the skin/hair or the absence of pigment in the melanocytes or hairs. In dogs, true albinism is rare and is due to *SLC45A2* or *OCA2* mutations [1,2,3,4]. The absence of melanocytes in the skin, also called piebaldism or leucism, is a variable phenotype ranging from white spots to more extreme white patterns, resulting in almost-white coat color with few pigmented areas. These white spotting phenotypes involve different variants in the *MITF* or *KIT* gene [5,6,7,8]. On the other hand, uniformly white or cream solid coat color in dogs, due to the absence of pigment or minimal pigment, was suggested to originate from dilution of the phaeomelanin, either to an extremely pale cream or to a white color. Such dogs are not albino and retain pigmentation in their nose, leather, eye rims, lips, or pads. In 1957, Little suggested the involvement of the C locus [9]. Further, Sponenberg and Rothschild suggested that the solid white phenotype would result from both the expression of only phaeomelanin and its extreme dilution at a locus I (Intensity) [10]. This hypothesis was then consolidated by Schmutz and Berryere showing that some solid white dogs have a non-functional *MC1R* gene (*e*/*e* genotype) and only express phaeomelanin [11]. However, the precise localization and the gene involved in locus I is not known. In the present study, we used a multiple breed approach to identify the genetic basis of the solid white coat color involving extreme phaeomelanin dilution in dogs. Based on several genome-wide association studies (GWAS), first using solid white dogs and non-white dogs from five breeds (White Shepherds, German Shepherds, Poodles, Cotons de Tulear and Bichons), and second, using 15 other breeds presenting phaeomelanin dilution, we identified the I locus on chromosome 20 and a missense variant linked to phaeomelanin dilution in dogs.

## 2. Materials and Methods

### 2.1. Sample Collection

Blood and tissue biopsy samples from dogs and wolves were collected by a network of veterinarians through the Cani-DNA BRC (http://dog-genetics.genouest.org) and DNA/RNA were extracted as previously described [12]. The work with dog samples was approved by the CNRS ethical board France (35-238-13). The blood and tissue samples were done by veterinaries along medical cares of dogs: blood and tissue were collected at medical visit or at surgery, then stored in EDTA or RNAlater tubes, respectively.

### 2.2. Genome-Wide Association Study (GWAS)

DNAs were genotyped on the Illumina 170 k SNV array and on Affymetrix 712 k SNV array at CNG (Evry, France) and Affymetrix, Inc. (Santa Clara, CA, USA) respectively. We used mixed linear model analyses, taking into account population structure and kinship (Eigenstrat), removing SNVs and individuals with a call rate below 95%.

For imputation, the MACH software [13] was used to impute 444 variants identified from the whole genome sequencing of the 577 dogs from the Dog Biomedical Variant Database Consortium (DBVDC). The default settings of MACH software were used (with following option --rounds 50 --states 200 --mask 0.02).

### 2.3. Filling Gap in the CanFam3 Sequence of MFSD12

To fill the gap (Chr20:55,840,001-55,870,000) containing the first exon 1 of the *MFSD12* locus, long read sequences were produced through MinION (Oxford Nanopore Technologies) according to manufacturer instructions. Reads were mapped to the dog genome with minimap2 [14] using default parameters (-ax map-ont parameter) and reads overlapping the gap were selected to fill the gap. Selected long reads were used as targets to map the unmapped canine reads from whole-genome sequences Illumina short reads of DBVDC [15] (File S1).

### 2.4. Sequencing and RT-PCR on MFSD12

Sanger sequencing of DNA canine samples was performed as previously described [12]. The qPCR was performed on cDNA samples diluted 1:20 with the SYBR Green PCR master mix on the 7900HT Fast Real-Time PCR System (Applied Biosystems, Foster City, CA) using standard procedures. Each PCR was carried out in triplicate. Relative amounts of the transcript were determined using the delta Ct method. The mRNA levels for each genotype were calculated as a fold increase compared with the canine housekeeping *HPRT* gene (ENSCAFG00000018870) in skin, for 6 homozygous mutant dogs, 6 heterozygous dogs and 3 homozygous wild type dogs. Primers are available in Supplementary Table S1.

## 3. Results

### 3.1. A Novel Association With Solid White on Canine Chromosome 20.

**Figure 1:**
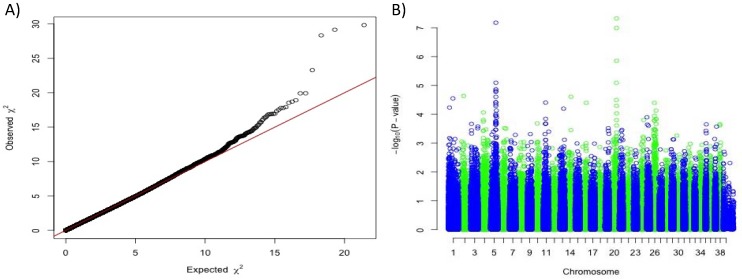
Genome-wide association study results based on 34 white dogs (White Shepherds, Poodles, Cottons de Tulear and Bichons) versus 128 non-white dogs (German Shepherds and Poodles). (**A**) Quantile-Quantile plot displaying a genomic inflation λ of 1.000015, indicating no residual inflation. (**B**) Manhattan plot displaying the results from the GWAS. This analysis pointed out two loci on chromosome 5 (Chr5:63,666,161 *p*_corrected_ = 6.7 × 10^−8^) and on chromosome 20 (Chr20:55,213,866, *p*_corrected_ = 4.7 × 10^−8^).

Using a panel of 34 white dogs from four different breeds (7 White Shepherds, 7 Poodles, 5 Cotons de Tulear and 15 Bichons) and 128 non-white dogs (24 German Shepherd Dogs and 104 Poodles), we first performed a genome-wide association study (GWAS) correcting for population stratification and cryptic relatedness. We detected two significant associations with solid white: one on chromosome 5, 28 kb upstream from the *MC1R* gene (CanFam3.1 position 64 Mb; *p*_raw_ = 5.3 × 10^−12^, *p*_corrected_ = 6.7 × 10^−8^), and a novel locus on chromosome 20 (position 55 Mb; *p*_raw_ = 9.13 × 10^−12^, *p*_corrected_ = 4.7 × 10^−8^) (Figure 1).

The association with the *MC1R* locus was hypothesized to be due to the *e* allele and the exclusive production of phaeomelanin in solid white dogs, as previously shown [11]. To confirm this hypothesis, we checked the genotypes at *MC1R*:c.916C > T responsible for the *e* allele in the 577 dog whole-genome sequences from the Dog Biomedical Variant Database Consortium (DBVDC) [15] and confirmed that out of 26 solid white dogs from West Highland White Terrier, Bichon and White Shepherd breeds, all were homozygous *e*/*e* for the *MC1R* allele (Fisher exact test *p*-value = 1.36 × 10^−26^). Thus, solid white dogs do not express eumelanin but only phaeomelanin, and we hypothesized that this phaeomelanin is diluted by a variant allele located on the novel chromosome 20 locus.

### 3.2. Identification of a Coding MFSD12 Variant Linked to Phaeomelanin Dilution

**Figure 2:**
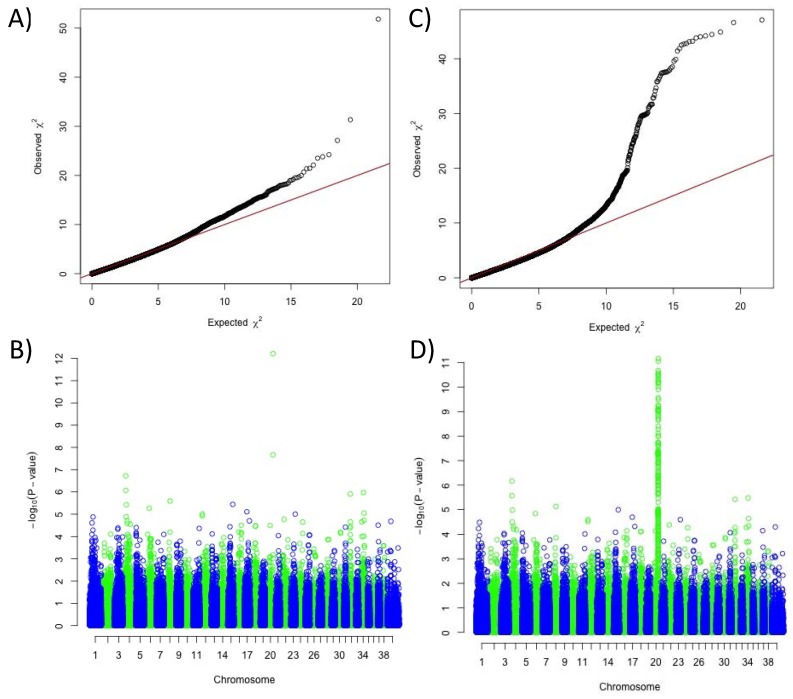
Results of the genome-wide association study for the phaeomelanin dilution phenotype, using 138 cases (dogs with diluted phaeomelanin) and 2325 controls (dogs with undiluted phaeomelanin). (**A**) Quantile-Quantile plot of GWAS displaying a genomic inflation λ of 1.000039. (**B**) Manhattan plot displaying the results from the GWAS: two significant SNVs on chromosome 20 (chr20:55,850,145, *p*_raw_ = 5.5 × 10^−289^, *p*_corrected_ = 6.01 × 10^−13^ and chr20:55,213,866, *p*_raw_ = 1.26 × 10^−175^, *p*_corrected_ = 2.16 × 10^−8^). (**C**) Quantile-Quantile plot of GWAS with imputed SNVs from the candidate region of chromosome 20 displaying a genomic inflation λ of 1.000045. (**D**) Manhattan plot displaying the results from the GWAS with imputed SNVs from the candidate region of chromosome 20: a unique highly significant locus on chromosome 20 (two best SNVs Chr20:55,850,145, *p*_raw_ = 5.5 × 10^−289^, *p*_corrected_ = 6.82 × 10^−12^ and chr20: 55847284, *p*_raw_ = 15.08 × 10^−286^, *p*_corrected_ = 8.74 × 10^−12^), separated by 2 kb.

**Figure 3:**
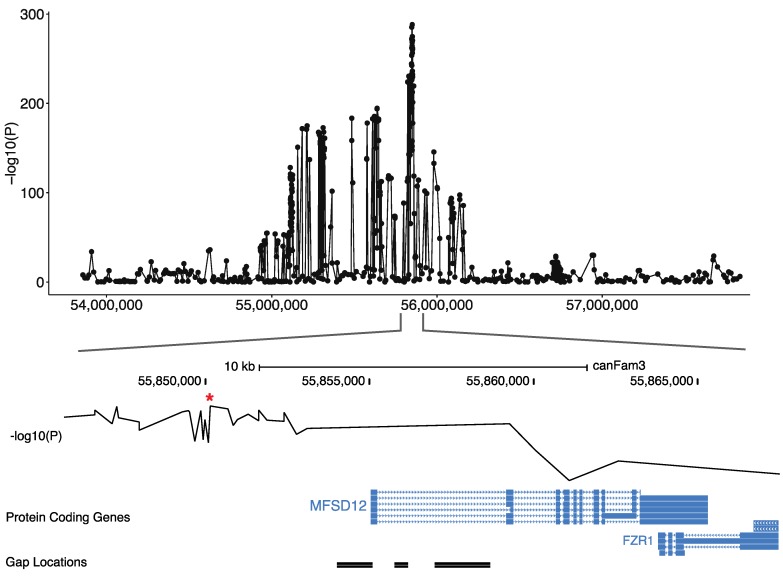
Screenshot of the locus on the canine chromosome 20 locus associated with the phaeomelanin dilution. The GWAS p-values of the 444 imputed variants on the 138 cases and 2325 controls are represented on the top. The best GWAS SNV (indicated by a *) located ~5 kb upstream of the *MFSD12* gene is represented on the middle and the *MFSD12* exon position, relatively to the gap position is illustrated on the bottom.

Based on the assumption that the solid white is a combination of two mechanisms: the expression of only phaeomelanin (*e*/*e* at *MC1R*) and the extreme phaeomelanin dilution (new locus on chromosome 20), we decided to complete our GWAS panel by adding breeds presenting with fixed diluted or undiluted phaeomelanin. Combining markers from the Illumina SNV chip and SNVs extracted from the 577 dog whole-genome sequences (DBVDC) [15], we performed a novel multi-breed GWAS using 138 cases (breeds with fixed diluted phaeomelanin) and 2325 controls (breeds with fixed undiluted phaeomelanin) (Table S2). We detected a significant association with solid white on the previously identified locus of chromosome 20, with a higher *p*-value; the best SNV being located ~5 kb upstream of *MFSD12* gene (CanFam3.1 position on Chr20: 56 Mb, *p*_raw_ = 5.5 × 10^−289^, *p*_corrected_ = 6.01 × 10^−13^) (Figure 2). Variants in this gene have recently been associated with skin pigmentation in humans [16,17] and grey coat color in mice [17] resulting from dilution of the phaeomelanin pigment with normal eumelanin production. In addition, variants of this gene have also been associated with the mushroom coat color dilution in ponies [18]. Thus, to identify the causal variant linked to the phaeomelanin dilution in dogs, we imputed 444 variants, identified through the 577 dog sequencing data (DBVDC) [15], spanning 2 Mb (chr20:54.8–56.8 Mb) on the chromosome 20 locus. This imputation did not allow the identification of a most significant SNV (Figure 2 and Figure 3). Since the best associated SNV was located ~5 kb upstream of the *MFSD12* gene, we hypothesized that this variant could affect *MFSD12* expression. By quantitative RT-PCR, we showed that *MFSD12 mRNA* expression in the skin does not differ in dogs with or without this variant genotype, and so we thus ruled out this hypothesis (Figure S1).

To identify the causal variant, we further searched for gaps of the CanFam3 genome assembly in this region and identified a gap harboring the first exon of the *MFSD12* candidate gene (Figure 3). Long read sequences of a poodle were produced to fill this gap and the DBVDC resource [15] was used to identify potential variants. Mapping the unmapped reads from whole-genome sequences of a white dog (West Highland White Terrier) and a non-white dog (Australian Cattle Dog) from the DBVDC [15], we identified a unique missense variant that was located in the first exon of *MFSD12* (File S1), based on alignments with the human genome and the annotation of the human orthologous transcript ENST00000355415: *MFSD12*: p.(Arg51Cys). This dog variant is exactly orthologous to the human variant rs751585493 (ENST00000355415 Chr19:3557253 G > A; p.(Arg51Cys). which is rare in humans with an allele frequency of 1.38390e-05 across all populations in gnomAD exome database, and only found in South Asian populations. A damaging effect of this variant (C > T) is predicted by most prediction tools, with scores such as 0.02 for SIFT (Damaging) and 0.806 for Polyphen (Probably damaging) [19].

### 3.3. Validation of the MFSD12 Coding Variant

**Figure 4:**
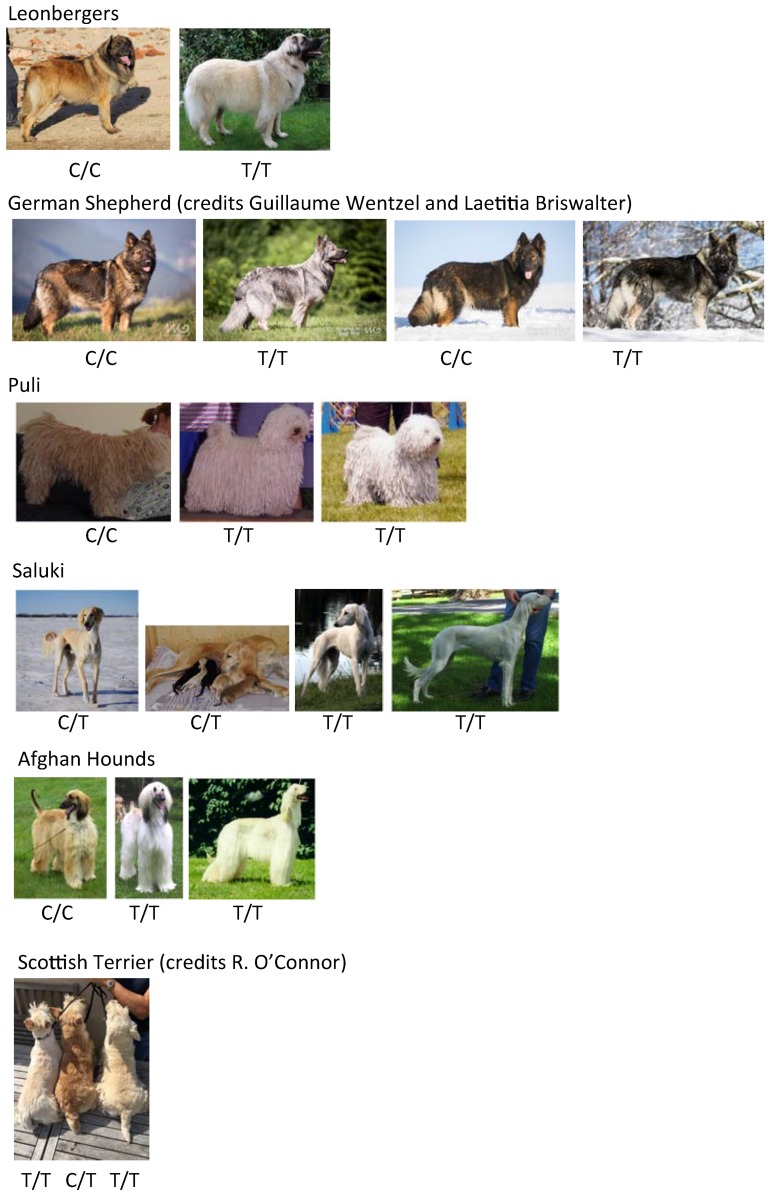
Photos and *MFSD12* genotypes of relevant tested breeds.

To confirm the association between this *MFSD12* coding variant and the phaeomelanin dilution phenotype, we checked for the presence of this variant in at least eight dogs from breeds with a fixed dilution of phaeomelanin (Pug, West Highland White Terrier, Bichons, White Shepherd, Alaskan Malamute). We confirmed that the variant perfectly segregates with breeds presenting diluted phaeomelanin but not with breeds presenting the fixed red coat color, using Irish Setter dogs as controls. For the following breeds thought to be fixed for the phaeomelanin dilution, out of eight tested dogs, the variant was very frequent: Schnauzer (94.4%), Husky (93.8%), Samoyed (87.5%), Akita (56.3%), Coton de Tulear (50.0%). We also tested Poodles, German Shepherds, and Leonbergers, using dogs presenting various shades of phaeomelanin dilution. We confirmed the enrichment of the *MFSD12* variant in dogs presenting an extreme dilution of phaeomelanin to white, with 64 cases and 235 controls (*p*-value= 1.35 × 10^−53^, Chi2 test) (Table 1). None of the German Shepherd Dogs or Leonbergers had an *e*/*e* genotype for *MC1R.* This was also observed in Caucasian Mountain Dog (2 dogs), Puli (3 dogs), Saluki (4 dogs) and Afghan Hound (3 dogs) (Figure 4). For example, the black mask caused by an *E^M^* allele at *MC1R* was not diluted in the two shown Leonbergers (Figure 4). The solid white coat color is not a simple monogenic trait since homozygous dogs for the variant *MFDS12* allele, while they are paler than other dogs of the same breed, do not always have extreme white coat color, as observed for Scottish Terriers or Leonbergers (Figure 4).

This *MFSD12* variant appears to be ancient since it was found in nine wolves (arctic wolves and grey wolves from Canada or Poland) of the 31 tested wolves (unspecified European, Siberian, Mongolian, Canadian wolves). Wolves do not carry an *e*/*e* genotype, but instead are wild type (*E/E*) at *MC1R*. In these wolves, the eumelanin is still present in their coat as intermingled black hairs or black-tipped hairs, but the phaeomelanin is diluted to cream when the *MFSD12* allele is homozygous.

## 4. Discussion

Thanks to a multiple step GWAS approach, we confirmed that solid white coat color in dogs may result from the absence of eumelanin (*e*/*e* at the *MC1R* locus) and an extreme dilution of phaeomelanin, confirming the hypothesis of Sponenberg and Rothschild on the existence of an *I* locus and later, the work of Schmutz and Berryere [10,11]. Although an *e*/*e* genotype at *MC1R* and an *i*/*i* genotype at *MFSD12* causes cream to white in many dog breeds, it is probable that other modifier alleles exist and explain the cream to white dilution in dogs. Benefiting from the fixed coat colors in many canine breeds, a multiple breed mapping approach allowed us to identify the gene and variant underlying the *I* locus, by the discovery of a variant significantly linked to the dilution of phaeomelanin. After the identification of a strongly significant association between chromosome 20 and the fixed white coat color in specific breeds, we used publicly available dog genomes (DBVDC resource) [15] and targeted long read sequencing to narrow down the search of the candidate variant. These experiments allowed us to fill a gap of the Canfam3.1 dog sequence, which was crucial to identify a coding variant in the first exon of *MFSD12* linked to phaeomelanin dilution. The finding of only one coding variant in the *MFSD12* sequence and the fact that this variant is predicted as deleterious by most prediction tools are strong arguments to point out this variant as causal. In addition, we checked if the mRNA expression of *MSD12* was decreased in dogs presenting the variant compared to wild type dogs and did not find any significant change, suggesting that the variant affecting *MSFD12* is probably not a regulatory variant. All these arguments suggest that the coding variant in the first exon of *MFSD12* causes the phaeomelanin dilution. However, considering the high linkage disequilibrium (LD) at the locus, one should be careful not to rule out the involvement of another SNV (in LD with the best GWAS SNV) in phaeomelanin dilution. Although we did not observe any significant difference between the expression levels of wild-type versus mutated *MFSD12* mRNAs, we cannot rule out other posttranscriptional or epigenetic alterations leading to non-functional MFSD12 protein. Thus, further functional studies are needed to confirm the role of this coding variant in dogs and humans. Finally, this variant is probably ancient since it was detected in many genetically diverse breeds, old breeds, and even wolves. Further studies are needed to see if this variant is under natural selection in wolves and correlated with latitude.

Looking at human pigmentation data, *MFSD12* was recently associated with diluted skin pigmentation [16,17] and since we found an exactly homologous variant of *MFSD12* (rs751585493) in the human database, in South Asian populations we anticipate that this SNV is also associated with phaeomelanin dilution in humans, enriching the list of variants linked to skin pigmentation in mammals. The *MFSD12* gene was, very recently, also associated with the Mushroom coat color in horses [18] and with the grey coat color in mice [17], resulting from dilution of the phaeomelanin pigment with normal eumelanin production. Functional analyses indicate that MFSD12 is involved in lysosomal biology but not in eumelanosomes [16,17], answer the question of why eumelanin is not affected by these variants. Altogether, the present data, as well as data from the literature, confirm that *MFSD12* plays a conserved role in phaeomelanin intensity and vertebrate pigmentation.

## Figures and Tables

**Table 1:** Genotyping of *MFSD12* variant in Poodle, Old German Shepherd and Leonberger breeds showing enrichment of the variant in dogs with phaeomelanin dilution.

Breed	Coat Color	T/T	T/C or C/C
Poodle *	white	8	0
	apricot/cream/red	2	57
Old German Shepherd **	white phaeomelanin	44	5
	red or sable phaeomelanin	4	54
Leonberger ***	diluted phaeomelanin	7	0
	fawn sable	0	118
All 3 breeds ****	diluted phaeomelanin	59	5
	red or sable phaeomelanin	6	229

* *p*-value = 6.9 × 10^−9^ (Exact test of Fisher), ** *p*-value = 1.22 × 10^−19^ (Exact test of Fisher); *** *p*-value = 1.25 × 10^−11^ (Exact test of Fisher); **** *p*-value = 1.35 × 10^−53^ (Chi2 test).

## Supplementary Materials

The following are available online at https://www.mdpi.com/2073-4425/10/5/386/s1, Figure S1: RT-qPCR experiments on *MFSD12* according to Chr20:55850145 genotype, Table S1: Primers used in this study for qPCR experiments and variant sequencing, Table S2: Dogs breeds selected for the GWAS, File S1: The sequence of the gap at chr20:55854007-55855102.
