# Supplementary material for: Identification of a Missense Variant in MFSD12 Involved in Dilution of Phaeomelanin Leading to White or Cream Coat Color in Dogs
Source: Genes (Basel). 2019 May 21;10(5):386. doi: 10.3390/genes10050386 (PMC6562630; doi:10.3390/genes10050386)
Supplement: Supplementary file 1 [file genes-10-00386-s001.pdf]

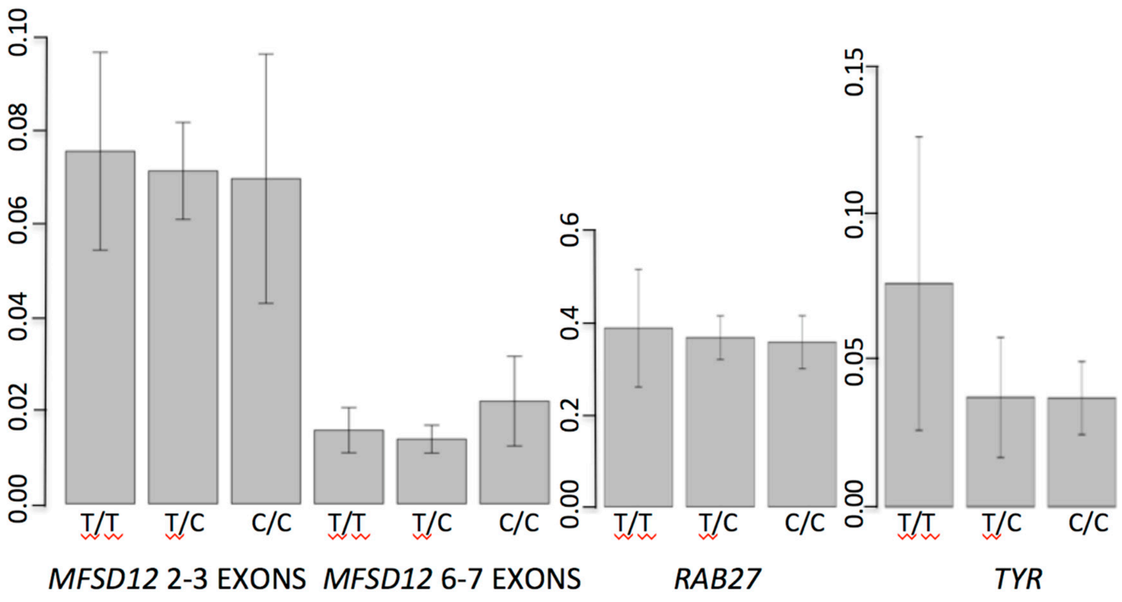

Figure S1. RT-qPCR experiments on *MFSD12* according to Chr20:55850145 genotype. The *MFSD12* expression level does not significantly differ between the skin of 6 homozygously mutated dogs (T/T) or 6 heterozygous dogs (T/C) and 3 homozygous wild type dogs (C/C). Expression of *Rab27* and *Tyr* genes were also tested as reference genes of melanocyte and thus expected to be invariable.

Table S1. Primers used in this study for qPCR experiments and variant sequencing.

Target	strand	Sequences	Purpose
MFSD12 : exon 2-3	Forward	TCATTGTCATCTTCCAGTTCGG	qPCR
	Reverse	ACCACGGTGAAGGCATACC	
MFSD12 : exon 6-7	Forward	GAGACACTGGCTGCGGGAG	qPCR
	Reverse	GAGTAGGTGAGGTACATGGCTA	
TYR	Forward	TGAGTACATGGGAGGGCG	qPCR
	Reverse	CTGTCGGCTGTTGTACTCCT	
RAB27	Forward	GTGGGCATTGATTTCAGGGA	qPCR
	Reverse	CCCTGCTGTGTCCCATAATTG	
HPRT	Forward	CGCTGAGGATTTGGAAAAAG	qPCR
	Reverse	AGAGGGCTACGATGTGATGG	
SNV chr20:55850145	Forward	AACACAAGTCTCGcacacac	Sequencing
	Reverse	CCTGATCCTACCCCGTGTTG	
Exon1 of *MFSD12* variant	Forward	CTTCCTCAATGACCTGTGCG	Sequencing
	Reverse	CCGCGACTTACCAACCAG	

Table S2. Dogs breeds selected for the GWAS to identify genes involved in phaeomelanin dilution using dogs belonging breeds with undiluted phaeomelanin and to breeds with fixed undiluted phaeomelanin.

	Breeds	Number
Control breeds	Affenpinscher	1
	Airedale Terrier	2
	Alpine Dachsbracke	1
	American Bulldog	2
	American Staffordshire Terrier	1
	Appenzeller Sennenhund	18
	Australian Cattle Dog	5
	Australian Shepherd	4
	Australian Terrier	1
	Basenji	6
	Basset Hound	3
	Bavarian Hound (Bayerischer Gebirgsschweisshund)	1
	Beagle	16
	Beauceron	19
	Belgian Shepherd Dog	12
	Bernese Mountain Dog	572
	Border Collie	208
	Border Terrier	26
	Boxer	44
	Brittany Spaniel	32
	Brussels Griffon	3
	Bull Terrier	3
	Bullmastiff	2
	Cane corso	1
	Cavalier King Charles Spaniel	6
	ChowChow	3
	Cocker Spaniel	17
	Coton de Tulear	1
	Curly Coated Retriever	1
	Dachshund	19
	Dalmatian dog	3
	Doberman Pinscher	28
	Dogue de Bordeaux	301
	Dutch Shepherd	1
	English Cocker Spaniel	1

	English Pointer	4
	English Setter	38
	Entlebucher Sennenhund	16
	Flat Coated Retriever	33
	Friesian Stabyhoun	2
	German Shepherd	51
	German Shorthaired Pointer	5
	German Wirehaired Pointer	1
	Gordon Setter	31
	Grand Anglo Francais	10
	Great Dane	1
	Greater Swiss Mountain Dog	206
	Heideterrier	1
	Hovawart	1
	Irish Setter	3
	Irish Terrier	35
	Italian Greyhound	1
	Jack Russell Terrier	14
	Jagdterrier	2
	Karelian Bear dog	1
	King Charles Spaniel	11
	Kromfohrlander	1
	Kunming Dog	9
	Landseer	1
	Leonberger	51
	Malinois	6
	Miniature Bull terrier	1
	Newfoundland	2
	Norwich Terrier	4
	Nova Scotia Duck Tolling Retriever	24
	Papillon	3
	Pembroke Welsh Corgi	1
	Pinscher	8
	Poodle	111
	Rhodesian Ridgeback	4
	Rottweiler	120
	Scottish Terrier	1
	Shetland Sheepdog	2

	St. Bernard	2
	Staffordshire Bull Terrier	1
	Tibetan Mastiff	10
	Vizsla (smooth coat)	2
	Weimaraner	2
	Welsh Springer Spaniel	3
	Yorkshire Terrier	125
Case breeds	Alaskan Husky	2
	Alaskan Malamute	2
	Bichon	8
	Bichon Frise	7
	Irish Soft Coated Wheaten Terrier	1
	Maltese dog	5
	Poodle	6
	Pug	19
	Samoyed	3
	Schnauzer	50
	Siberian Husky	3
	Silky Terrier	1
	West Highland White Terrier	22
	White Shepherd	9

File S1. The sequence of the gap at chr20:55854007-55855102. In yellow, the first exon of *MFSD12*, according the to human orthologous transcript ENST00000355415; in brackets, the missense variant is shown.

CCGCAGGCAGCCCCCCGCCCACCCTCCACGCCCCCGGCCCACCCCGTACCCCCGCCCCCGGCCCCACTCATTA

GGCCTCGTGTAGCAGCGGGCGGGCTGGGGCGGCGCGGCCCGGAGGCCGGAGTGTGGGTGTGGGCACCCCCGCGCCGTCCC

CCGCCCCTGGGGGCCCGCGAGCCCACGGTGAGCCGTCGGGCGCTAGGACCGCCCCGCCCCGCCGGCGCAGGGCGTGGAGG

CCGCGCTGGCCCCGAGGCCACGCCCCCTCTCCGGTGATTGGCTGCCGGGCTCCGGCGCGCGGCCCCGATTGGCGGGGGCG

GCCGCCAGCATCCCCCGCCCCGCCCCGCCCGGGCCTTAATGCGGGGTTCGGGGCCCGGCGGCCGCAGGCGCGCTCGGAGG

GGCGGGCTTGGGCGGCGGCGGCGCGGGGAGAGCCGGGCGGGGACGGTGCGCCCCGGGGTCGCGTCTGCCGCTCGCCAGAC

CGCCCGGCCCGGGTGCGGAGCGCGGCCATGGGCCCCGGGCCCCCGGCGGCCGGAGCGGCGGCGCCCCCGCGGCCCCTGTC

GCTGTCCGCGCGGCTGAGCTACGCGGTGGGCCACTTCCTCAATGACCTGTGCGCGTCCATGTGGTTCACCTACCTGCTGC

TCTACCTGCACTCGGTG [**C/T**] GCGCCTACAGCTCGCG
